# 

**Published:** 1894-06

**Authors:** 


					﻿NOTICE
All articles ^rpublicati^^^woks for^J^eu, buiineti communications, etc.,
must be eent to Editor 6^31 Locust t^eJf Philadelphia.
Subscribers will ^gn^^notice tfA^^Hns Journal will be sent until arrears
are paid and it is	discont^^9S.
				

